# Efficient and stable hybrid perovskite-organic light-emitting diodes with external quantum efficiency exceeding 40 per cent

**DOI:** 10.1038/s41377-024-01500-7

**Published:** 2024-06-12

**Authors:** Lingmei Kong, Yun Luo, Qianqian Wu, Xiangtian Xiao, Yuanzhi Wang, Guo Chen, Jianhua Zhang, Kai Wang, Wallace C. H. Choy, Yong-Biao Zhao, Hongbo Li, Takayuki Chiba, Junji Kido, Xuyong Yang

**Affiliations:** 1 Key Laboratory of Advanced Display and System Applications of Ministry of Education, Shanghai University, Shanghai, 200072 China; 2 Institute of Nanoscience and Applications, Department of Electrical and Electronic Engineering, Southern University of Science and Technology, Shenzhen, 518055 China; 3 Department of Electrical and Electronic Engineering, The University of Hong Kong, Hong Kong, China; 4 Department of Physics and Lakeside AR/VR Laboratory, International Joint Research Center for Optoelectronic and Engineering Research, Yunnan University, Kunming, 650091 China; 5 Experimental Center of Advanced Materials, School of Materials Science and Engineering, Beijing Institute of Technology, Beijing, 100081 China; 6 Graduate School of Organic Materials Science, Frontier Center for Organic Materials, Yamagata University, 4-3-16 Jonan, Yonezawa, 992–8510 Japan

**Keywords:** Lasers, LEDs and light sources, Displays

## Abstract

Light-emitting diodes (LEDs) based on perovskite semiconductor materials with tunable emission wavelength in visible light range as well as narrow linewidth are potential competitors among current light-emitting display technologies, but still suffer from severe instability driven by electric field. Here, we develop a stable, efficient and high-color purity hybrid LED with a tandem structure by combining the perovskite LED and the commercial organic LED technologies to accelerate the practical application of perovskites. Perovskite LED and organic LED with close photoluminescence peak are selected to maximize photon emission without photon reabsorption and to achieve the narrowed emission spectra. By designing an efficient interconnecting layer with p-type interface doping that provides good opto-electric coupling and reduces Joule heating, the resulting green emitting hybrid LED shows a narrow linewidth of around 30 nm, a peak luminance of over 176,000 cd m^−2^, a maximum external quantum efficiency of over 40%, and an operational half-lifetime of over 42,000 h.

## Introduction

Light-emitting diodes (LEDs), one of core parts of display equipment, are required to fulfill the requirements of Rec. 2020 color gamut standard for the next-generation display applications. Metal-halide perovskites have extremely narrowband emissions (full-width-at-half-maximum, FWHM ≤ 20 nm) and easily tunable bandgap (emission range: 410–850 nm), which are the only a few kinds of emitters that can fully satisfy the Rec.2020 so far^1–8^. The fast advances in perovskite LEDs (PeLEDs) have been made in the past few years and the current external quantum efficiency (EQEs) of PeLEDs have approached 30%^9–19^, comparable to those of commercial organic LEDs (OLEDs). However, the intrinsic instability of perovskites still casts a shadow over their practical applications^20–23^.

Combining highly color-purity perovskite emitters with other mature display technologies such as OLEDs to expand the display color gamut could be a shortcut towards commercialization for high-definition displays. Perovskite emitters on the other hand can also be deposited by thermal evaporation in vacuum, which is well compatible with production lines of OLEDs^24–27^. Thus, taking advantage of existing OLED-related equipment and processing, the integration of perovskite and OLED will be a rational solution. Indeed, hybrid display technologies have already developed and may become the mainstream trend for display field in the future. For example, a quantum dot liquid crystal TV is fabricated by applying quantum dot patterned color filters and liquid crystal display^28,29^, and a white light LED is prepared by combining QLEDs with OLEDs^30^. Tandem LEDs that connect two or more electroluminescence (EL) units of LEDs through a unique interconnecting layer (ICL) can offer an effective way to integrate the different technologies for better display applications^31–34^. Another important advantage of tandem devices is that the operational lifetime can be dramatically improved due to the reduced current under the same luminance, which is especially beneficial for the PeLEDs.

In this work, hybrid perovskite-organic LEDs are proposed to provide an alternative route for accelerating commercialization of perovskite. Specifically, an efficient ICL consisting of 1,4,5,8,9,11-hexaazatriphenylene hexacarbonitrile (HAT-CN)/MoO_3_/(4,4′-bis(N-carbazolyl)-1,1′-biphenyl) (CBP) is demonstrated to show high transmittance and good electrical connection. And by combining with good light-coupling design, green emitting PeLED and OLED with similar photoluminescence (PL) peak are well connected, achieving remarkable device performance. Benefiting from the high color purity of PeLED, the emission linewidth of the hybrid LEDs can be effectively tuned and a narrow FWHM of only around 30 nm is obtained in the optimized devices. Meanwhile, the resulting LEDs also demonstrate high electroluminescence (EL) performance, with a peak EQE of 43.42%, a maximum luminance of 176,166 cd m^−2^, and a long half-lifetime of 42,080 h at an initial luminance of 100 cd m^−2^.

## Results

**Fig. 1:**
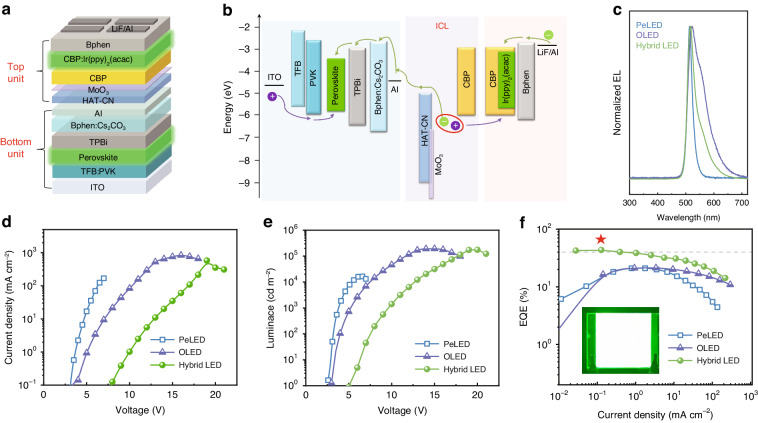
Device structure and performance. **a** Device structure and **b** schematic energy band diagram of a hybrid LED. **c** EL spectra, **d** *J–V*, **e** *L*–*V*, and **f** EQE-*J* characteristics of the PeLED, OLED, and hybrid LED. The star marks the maximum EQE value. Inset in (**f**) is a photo of a hybrid LED operated under biased voltage of 7.5 V, with an active area of 30 mm × 30 mm

The hybrid LED consists of a bottom PeLED and a top CBP:Ir(ppy)_2_(acac)-based OLED (Fig. 1a), which are vertically stacked and connected through an ICL that consists of HAT-CN as electron generation/separation layer, CBP as hole generation/separation layer, and MoO_3_ as the charge enhancement layer between HAT-CN and CBP. The energy band diagram of the hybrid device is shown in Fig. 1b, and the corresponding energy level values are from reported works^35^. As schematically illustrated, carriers are expected to efficiently generate at the interface of MoO_3_ and CBP as a result of the well-aligned energy levels of the proposed ICL and inject into the bottom PeLED and the top OLED under forward bias^36^. Note that the thicknesses for both the Al and MoO_3_ layers within devices are very thin. Unlike external Al electrode, the thin Al layer facilitates the electron separation and transport from the ICL. The ultra-thin MoO_3_ (only ~1 nm) allows holes to tunnel through the spacer, which plays an important role in increasing the concentration of charge carriers and extracting the more carriers. MoO_3_ has a deeper unoccupied lowest molecular orbital (LUMO) than that of HAT-CN, and meanwhile its LUMO is below the highest occupied molecular orbital (HOMO) of CBP that facilitates electron flow from p-type interface doped CBP to HAT-CN, thereby strengthening the build-in electric field and allowing us to obtain the high-performance devices.

The bottom PeLED with a structure of indium tin oxide (ITO)/poly(9,9-dioctyl-fluorene-co-*N*-(4-butylphenyl)diphenylamine) (TFB): poly(*N*-vinylcarbazole) (PVK)/perovskite/1,3,5-tris(1-phenyl-1H-benzimidazol-2-yl)benzene (TPBi)/4,7-diphenyl-1,10-phenanthroline (Bphen):Cs_2_CO_3_/Al and the top OLED with a structure of ITO/HAT-CN/MoO_3_/CBP/CBP:Ir(ppy)_2_(acac)/TPBi/LiF/Al were also fabricated for comparison. Figure 1c shows the normalized EL spectra of PeLED, OLED, and hybrid LED. The EL peaks of PeLED and OLED are around 514 nm and 523 nm, respectively, while the hybrid LED exhibits a peak centered at 516 nm dominated by the sharp emission linewidth of perovskite emission. Benefiting from the narrow EL emission profile, the hybrid LED shows a FWHM of only 31 nm, which is even less than half that of the OLED (67 nm), indicating its high color-purity. The performance of these proposed LEDs is displayed in Fig. 1d–f. The current density–voltage (*J*–*V*) and luminance–voltage (*L*–*V*) characteristics of the hybrid LED shows slow current injection as a result of a larger resistance induced by the multiple layers, and the luminance reaches 176,166 cd m^−2^, which is much higher than that of the single sub-unit devices (Fig. 1d, e). Notably, a very high peak EQE of 43.42% corresponding to a current efficiency (CE) of 156 cd A^−1^ (Fig. S1), almost the sum value of those of PeLED (21.07%) and OLED (21.33%), is obtained, suggesting the highly-efficient carrier generation, injection and recombination in the hybrid LEDs (Fig. 1f). The detailed EL performances are summarized in Table S1. The inset shows the bright green emission of hybrid LED devices at 7.5 V bias, with a large active area of 30 mm × 30 mm, demonstrating the great potential in large-area display application.

**Fig. 2:**
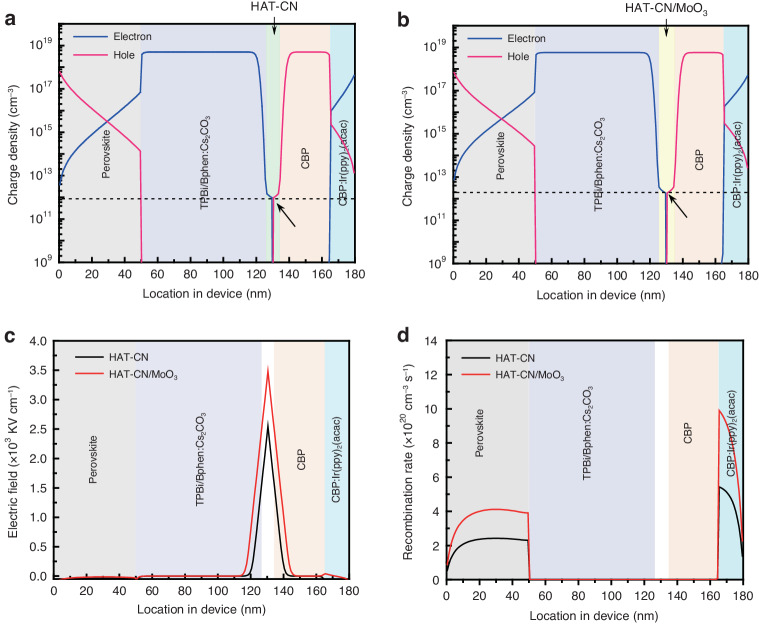
Simulation for hybrid LEDs. Distribution of charge density in the hybrid LED with **a** CGL and **b** m-CGL. Distribution of **c** electric field and **d** recombination rate in the hybrid LED with CGL and m-CGL

The performance of the hybrid device is closely related to the intermediate connector that should efficiently generate charges at its interface and smoothly inject charges into each sub-unit. To evaluate the optical and electrical properties of the proposed ICL, we investigated two ICLs with structures of HAT-CN/CBP and HAT-CN/MoO_3_ (1 nm)/CBP, which are denoted as the charge generation layer (CGL) and m-CGL, respectively. Theoretical analyses of charge density of the hybrid LEDs with CGL and m-CGL were conducted through simulation and calculations, where a well-established physical drift-diffusion model that consists of the continuity equation for electrons and holes coupled with the Poisson equation was used. Furthermore, field-dependent Miller-Abrahams theory was also considered to study the carrier transfer process between Bphen:Cs_2_CO_3_, CGL (or m-CGL) and CBP interfaces^37^. Figure 2a, b shows the distributions of charge density in the operating LEDs. For the hybrid LED with CGL, the charge densities of separated electrons and holes in HAT-CN/CBP are 9.53 × 10^11 ^cm^−3^, respectively. While in the hybrid LED with m-CGL, 1.9 times increase of the charge density (1.83 × 10^12 ^cm^−3^) is obtained at the interface of HAT-CN/MoO_3_/CBP, indicating a stronger generation and separation rate. The distribution of electric field was further analyzed in Fig. 2c. Due to the efficient charge carrier generation at HAT-CN/CBP interface as described above, electrons and holes are separated to form electric dipoles, thus yielding a build-in electric field. With the insertion of the ultra-thin MoO_3_, the separation of carriers is promoted, and the number of electric dipoles increases, leading to a notably enhanced electric field. We then present the distribution of the recombination rate in sub-units without/with MoO_3_. As shown in Fig. 2d, the recombination peaks are observed in both the perovskite and organic emitter layers, and it can be noted that the radiative recombination rate in whether perovskite or organic emitter can be significantly enhanced by 1.8 times when the ultra-thin MoO_3_ is deposited on HAT-CN. These simulation results indicate that the MoO_3_ layer facilitates charge carrier generation and separation from the ICL interface, and then transport smoothly via respective charge transport layers to make efficient exciton recombine in sub-units. We thus proposed that the charge generation capability can be strengthened when a MoO_3_ layer, with a deeper LUMO than that of HAT-CN, is introduced to HAT-CN/CBP interface. In this case, more electrons transferred from CBP to HAT-CN due to the existence of ultra-thin MoO_3_, and the hole concentration is increased in CBP, leading to higher hole current. Consequently, electrons and holes are injected into bottom PeLED and top OLED units, resulting in improved radiative recombination.

**Fig. 3:**
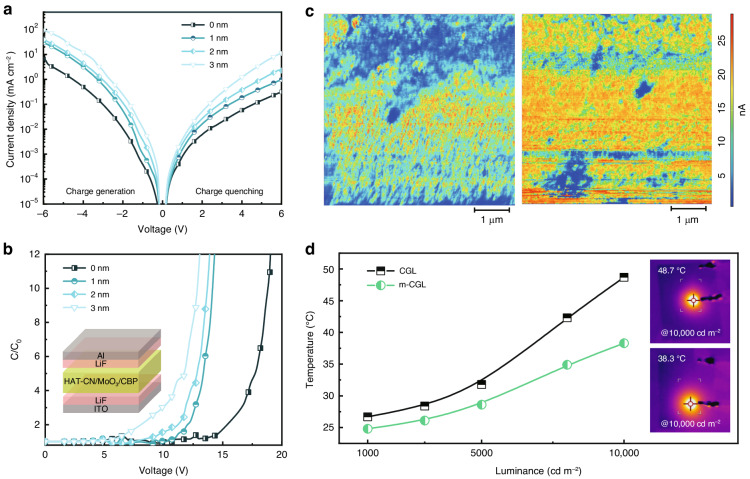
Electrical characteristics. **a** *J*–*V* and **b** *C*–*V* characteristics of the ICLs with varying MoO_3_ thickness from 0 to 3 nm. **c** c-AFM images based on ITO/HAT-CN (left) and ITO/HAT-CN/MoO_3_ (right). **d** Changes of surface temperature for PeLEDs with CGL and m-CGL

To investigate the effects of different thicknesses of MoO_3_ in the proposed ICL on the charge generation capability, the *J*–*V* curves of the ICL devices with a structure of ITO/HAT-CN/MoO_3_/CBP/Al as a function of MoO_3_ thickness varying from 0 to 3 nm were characterized. Under reverse bias, charge carriers are generated at the interface of HAT-CN/CBP and ultimately are extracted by the electrodes, whereas the charge carriers are injected from the electrodes and finally are quenched by the ICL under forward bias. If charge generation and charge quenching are identical, the ICL could generate charges efficiently in balanced fashion^38^. As shown in Fig. 3a, the symmetrical characteristics of *J–V* curves of the ICL devices are improved after inserting the thin MoO_3_, and the current density is continuously increased with increasing the thickness of MoO_3_, indicating that the charge generation and transport capacity are significantly enhanced. Moreover, the capacitance–voltage (*C*–*V*) characteristics of ICL devices were measured at a fixed frequency of 1 kHz. The device structure was shown in Fig. 3b, in which the double-insulating layers of LiF were introduced to inhibit the injection of charges from the external electrodes. The measured capacitance is normalized by the zero-bias capacitance (*C*_0_). The capacitance of the device without MoO_3_ show almost no change before an applied bias of 15 V, suggesting that a larger voltage is needed for the ICL to generate charge. In sharp contrast, for the m-CGL device the voltage required to generate charge is significantly reduced (7 V in 3 nm-MoO_3_ devices). Moreover, the ICL with a thicker MoO_3_ layer presents a superior electrical property in comparison to the one with a thinner MoO_3_ layer, consistent with the results of *J*–*V* measurements. The hybrid LEDs with different thicknesses of MoO_3_ were also fabricated and compared (Fig. S2 and Table S2). We found that the EL efficiency reaches the peak value when the thickness of MoO_3_ is at 1 nm and after that the EQE would gradually decreases. The efficiency decline is attributed to a decrease in transmittance of ICL that is detrimental to light outcoupling (Fig. S3) and a sharp increase in current density which can induce performance degradation due to exciton dissociation, Coulombic degradation and/or excessive heating^38^.

In order to clarify the effects of ultra-thin MoO_3_ on the electrical properties of ICL, the morphologies and conductibility of the HAT-CN films without/with MoO_3_ were investigated by atomic force microscopy (AFM) and conductive AFM (c-AFM). We noted that the HAT-CN films without and with depositing MoO_3_ have a similar roughness (Fig. S4), indicating that the surface morphology of the underlying HAT-CN is almost not affected by the ultra-thin layer of MoO_3_. However, this enables an enhancement in the electrical conductivity of HAT-CN, as revealed by c-AFM (Fig. 3c). From the line profiles of current (Fig. S5), we observed that the bottom of current profile is increased from 2 nA to 8 nA after the deposition of MoO_3_ on HAT-CN. X-ray photoelectron spectra (XPS) shows that MoO_3_ can partially diffused into the HAT-CN film (Fig. S6), thus improve electrical conductivity, which facilitates charge carrier transport of the ICL and suppresses undesired Joule heating due to the decreased resistance. As well known, Joule heating damages the operating stability of the device. We performed surface temperature tests on hybrid devices with CGL and m-CGL operating at different luminance (Fig. S7), and the temperature variation curves are shown in Fig. 3d. Both the devices show a comparable temperature of around 25 °C under a relatively low luminance of 1000 cd m^−2^. With the increase of applied voltage, the temperature difference between the two devices under the same luminance dramatically increases. Especially, the temperature of hybrid LEDs with CGL is 48.7 °C at 10,000 cd m^−2^, which is almost 1.3 times higher than that of hybrid LEDs with m-CGL (38.3 °C) at the same luminance. According to the rising trend of temperature, it can be predicted that the hybrid LEDs with m-CGL have a much lower surface temperature even at higher luminance. In addition to reducing heat in devices, the efficient charge transport and injection generated by the m-CGL greatly enhances the luminance. It is worth pointing out that achieving high luminance and efficiency at a low current density accompanied with low heat, is beneficial to long-term operational LEDs.

**Fig. 4:**
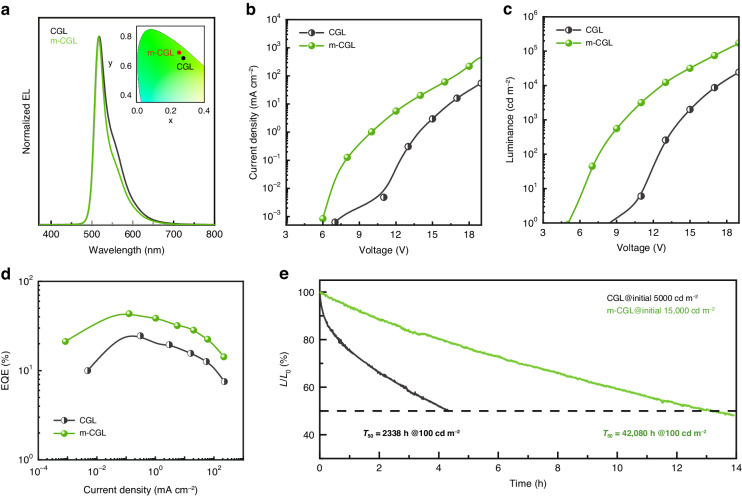
Performance comparison for the hybrid LEDs with different ICLs. **a** EL spectra, **b** *J–V*, **c** *L–V*, and **d** EQE-*J* characteristics, and **e** operational stability of the hybrid LEDs with CGL and m-CGL. Inset in (**a**) is the CIE coordinate of hybrid LEDs

The EL performance of hybrid LEDs based on CGL and m-CGL is then studied systematically (Fig. 4 and Table S3). Compared to the relatively wide EL FWHM (42 nm) of the LED with CGL, the FWHM of the device with m-CGL is dramatically reduced to 31 nm, and the Commission International de I’Eclairage (CIE) coordinates are accordingly changed from (0.28, 0.64) to (0.25, 0.67) that is closer to pure green light of (0.21, 0.71). The device with m-CGL exhibits faster current injection and higher current density characteristics, due to more efficient charge generation capability of ICL resulting from enhanced electron transfer at the HAT-CN/CBP interface, which is also evidenced by almost no obvious performance difference in the top OLED devices (Fig. S8 and Table S4). With the implementation of m-CGL, the EL performance of device is significantly improved: the luminance and the EQE are increased from 94,633 cd m^−2^ to over 176,000 cd m^−2^ and from 25.12% to over 43%, while the *V*_on_ is decreased from 8.5 to 5 V (Fig. 4d, e). The statistical histogram of the max. EQE values shows the average max. EQE values for these devices with CGL and m-CGL are 22.8 ± 5.5% and 39.9 ± 4.9%, respectively (Fig. S9), demonstrating good device-to-device reproducibility. It is noteworthy that the PeLEDs based on the perovskites with different systems show good compatibility with OLEDs in the hybrid device structures. We tested the operational stability of the hybrid LEDs based on quasi-2D/3D PeLED and OLED in the glove box without any encapsulation. The quasi-2D PeLED and OLED based hybrid LEDs with m-CGL show a half-lifetime (*T*_50_) of 13.2 h at an initial luminance (*L*_0_) of 15,000 cd m^−2^, while a 4.3 h *T*_50_ for the device with CGL at *L*_0_ of 5000 cd m^−2^ (Fig. 4e). By using the accelerated lifetime equation (${L}_{0}^{n}\,{T}_{50}$ = constant, where *n* is an acceleration factor)^39^ with *n* = 1.61 (Fig. S10), we therefore estimated the *T*_50_ of the hybrid LEDs with m-CGL at 100 cd m^−2^ to be 42,080 h, 18-fold improvement than that of the device with CGL. We also noted slight changes in EL spectra after the lifetime test or operated under different applied voltages (Fig. S11), which are ascribe to the relative instability of PeLEDs. Similarly, the hybrid LEDs with the combination of 3D PeLED and OLED shows significant stability improvement compared with the PeLED-only device, with a long *T*_50_ of 78,362 h (Figs. S12 and S13), demonstrating the potential of this hybrid structure on device performance.

## Discussion

In summary, we demonstrated highly efficient and stable hybrid LEDs with remarkable narrow linewidth emission constructed with a top OLED and a bottom PeLED by employing an efficient ICL based on a structure of HAT-CN/MoO_3_/CBP, in which the ultra-thin MoO_3_ sandwiched in the organic hole inject layer and hole transport layer not only facilitates the electron transfer from CBP to HAT-CN leaving a p-type doped HTL interface that leads to significant enhancement in the capacity for charge generation, separation and transport of the ICL, but also effectively suppresses the undesired Joule heating due to the decreased resistance. Benefiting from the proposed ICL and combining with good light outcoupling design, the hybrid LED exhibits high color purity, excellent device performance as well as significantly improved stability, representing one of the best performing tandem LEDs with ultra-high color purity to date. This work shows a great potential in the combination of the two or more display technologies for the practical applications, and the LEDs with high color purity, extremely high efficiency, and long operational lifetime would be ideal candidates to the next generation of full color displays and solid-state lighting markets.

## Materials and methods

### Materials

CsBr (99.999%, metal basis) and PbBr_2_ (99.999%, metal basis) were purchased from Alfa Aesar. Phenethylammonium bromide (PEABr) and PEDOT:PSS (Clevious PVP AI 4083) were purchased from Xian Yuri Solar Co, Ltd. PVK and dimethyl sulfoxide (DMSO) (99.9%) were purchased from Sigma-Aldrich. Cs_2_CO_3_, MoO_3_, TFB, Bphen, HAT-CN, CBP, Ir(ppy)_2_(acac), TPBi and LiF were purchased from Luminescence Technology Corp. All of the reagents were directly used as received.

### Preparation of perovskite films

The perovskite precursor solutions were prepared as previous reported with a modification^22,40^. Briefly, quasi-2D perovskite precursor was prepared by dissolving PbBr_2_, CsBr, and PEABr in anhydrous DMSO with the molar ratio of 1:1.2:0.4. 3D perovskite precursor was obtained by dissolving PbBr_2_, CsBr, and cesium trifluoroacetate in DMSO with the molar ratio of 1:1:0.7. The mixture was stirred at 60 °C for 3 h and filtered through a 0.45 μm polytetrafluoroethylene membrane before use. Perovskite films were deposited on glass or ITO substrates by spin-coating the precursor solutions at 4000 r.p.m for 40 s and annealing at 80 °C for 10 min.

### Device fabrication

TFB:PVK was deposited from its chlorobenzene solution (a weight ratio of 4:6, 10 mg mL^−1^) on the oxygen plasma treated ITO substrates at a speed of 4000 r.p.m. for 40 s, and baked at 120 °C for 20 min. For 3D PeLED, PEDOT:PSS as hole transport layer was spin-coated on the ITO substrates at 4000 rpm for 40 s and then baked at 150 °C for 20 min in air. After the deposition of the perovskite films, the samples were transferred into a high-vacuum deposition system (~4 × 10^−4 ^Pa) for the successive thermal evaporation of TPBi (40 nm), Bphen:Cs_2_CO_3_ (10 wt%, 40 nm), Al (1 nm), HAT-CN (10 nm), MoO_3_ (1 nm), CBP (35 nm), CBP:Ir(ppy)_2_(acac) (8 wt%, 15 nm), Bphen (65 nm), LiF (1 nm), and Al (100 nm).

### Device characterizations

Absorption spectra were recorded by using a PerkinElmer Lambda 950 UV-vis-NIR spectrometer. The surface temperature was measured by a FLIR T630sc thermal infrared imager. The *J*–*V* and *C*–*V* curves of the ICL devices were measured by using TTP4 cryogenic probe station. The *J*–*V* and *L*–*V* characteristics of LED devices were collected by using a QE Pro spectrometer and the Keithley 2400 source meter. The LED was tested on top of the integrating sphere, which has been accurately calibrated by a commercially standard LED. The operational lifetime tests of LEDs were performed on a commercialized ZJZCL-1 OLED ageing lifespan test system in a nitrogen-filled glovebox at room temperature. The electroluminescence performance for all the LED devices is measured based on an emission area of 2 mm × 2 mm.

### Simulation

Charge density, electric field, and recombination rate of proposed devices were simulated and quantitatively investigated by SETFOS 4.6, which was already successfully used to characterize organic solar cells^41,42^ and LEDs^43,44^. Here the well-established physical drift-diffusion model was used, which is consisted of the continuity equation for electrons and holes coupled with the Poisson equation. Furthermore, electron transfer process between HOMO and deep LUMO (e.g., electron transfer from the HOMO level of BCP to the LUMO of HAT-CN and MoO_3_) also considered to investigate carrier hopping process in devices. In addition, TPBi and Bphen:Cs_2_CO_3_ have been regarded as one “TPBi/Bphen:Cs_2_CO_3_” layer due to their approximate electron mobility and low energy barrier.

### Supplementary information

Supplementary Information for Efficient and stable hybrid perovskite-organic light-emitting diodes with external quantum efficiency exceeding 40 per cent
