# Supplementary material for: Efficient and stable hybrid perovskite-organic light-emitting diodes with external quantum efficiency exceeding 40 per cent
Source: Light Sci Appl. 2024 Jun 12;13:138. doi: 10.1038/s41377-024-01500-7 (PMC11169476; doi:10.1038/s41377-024-01500-7)
Supplement: Supplementary file 1 — Supplementary Information for Efficient and stable hybrid perovskite-organic light-emitting diodes with external quantum efficiency exceeding 40 per cent [file 41377_2024_1500_MOESM1_ESM.docx]

**Supplementary Information for**

**Efficient and stable hybrid perovskite-organic light-emitting diodes with external quantum efficiency exceeding 40 per cent**

Lingmei Kong^1^, Yun Luo^1^, Qianqian Wu^1^, Xiangtian Xiao^2^, Yuanzhi Wang^1^, Guo Chen^1^, Jianhua Zhang^1,^*, Kai Wang^2,^*, Wallace C. H. Choy^3^, Yong-Biao Zhao^4^, Hongbo Li^5^, Takayuki Chiba^6^, Junji Kido^6^ and Xuyong Yang^1,^*

^1^ Key Laboratory of Advanced Display and System Applications of Ministry of Education, Shanghai University, Shanghai 200072, China

^2^ Institute of Nanoscience and Applications, Department of Electrical and Electronic Engineering, Southern University of Science and Technology, Shenzhen 518055, China

^3^ Department of Electrical and Electronic Engineering, The University of Hong Kong, Hong Kong, China

^4^ Department of Physics and Lakeside AR/VR Laboratory, International Joint Research Center for Optoelectronic and Engineering Research, Yunnan University, Kunming 650091, China

^5^ Experimental Center of Advanced Materials, School of Materials Science and Engineering, Beijing Institute of Technology, Beijing 100081, China

^6^ Graduate School of Organic Materials Science, Frontier Center for Organic Materials, Yamagata University, 4-3-16 Jonan, Yonezawa, 992–8510 Japan

**
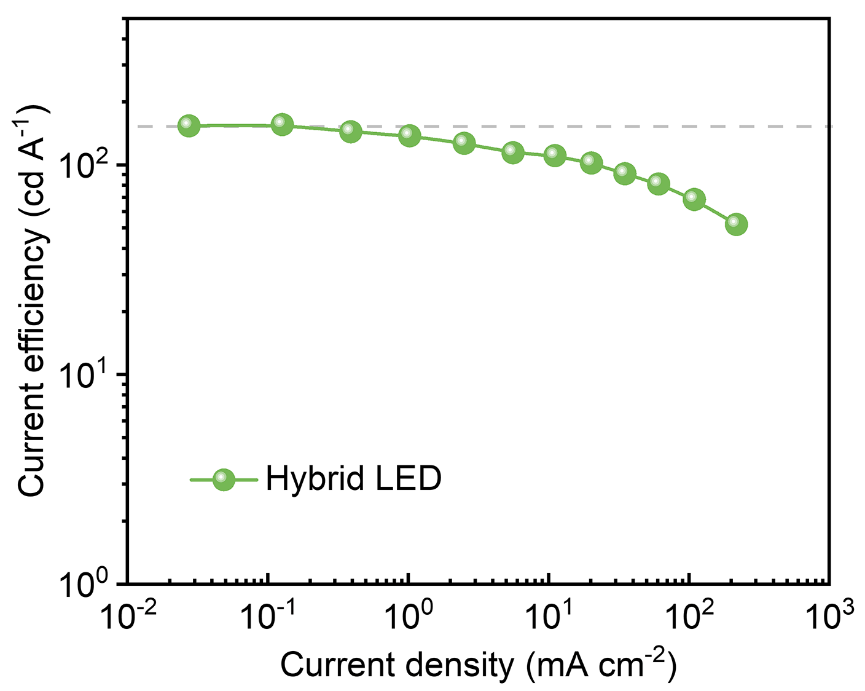
**

**Fig. S1.** CE-*J* characteristics of the hybrid LED with m-CGL.

**
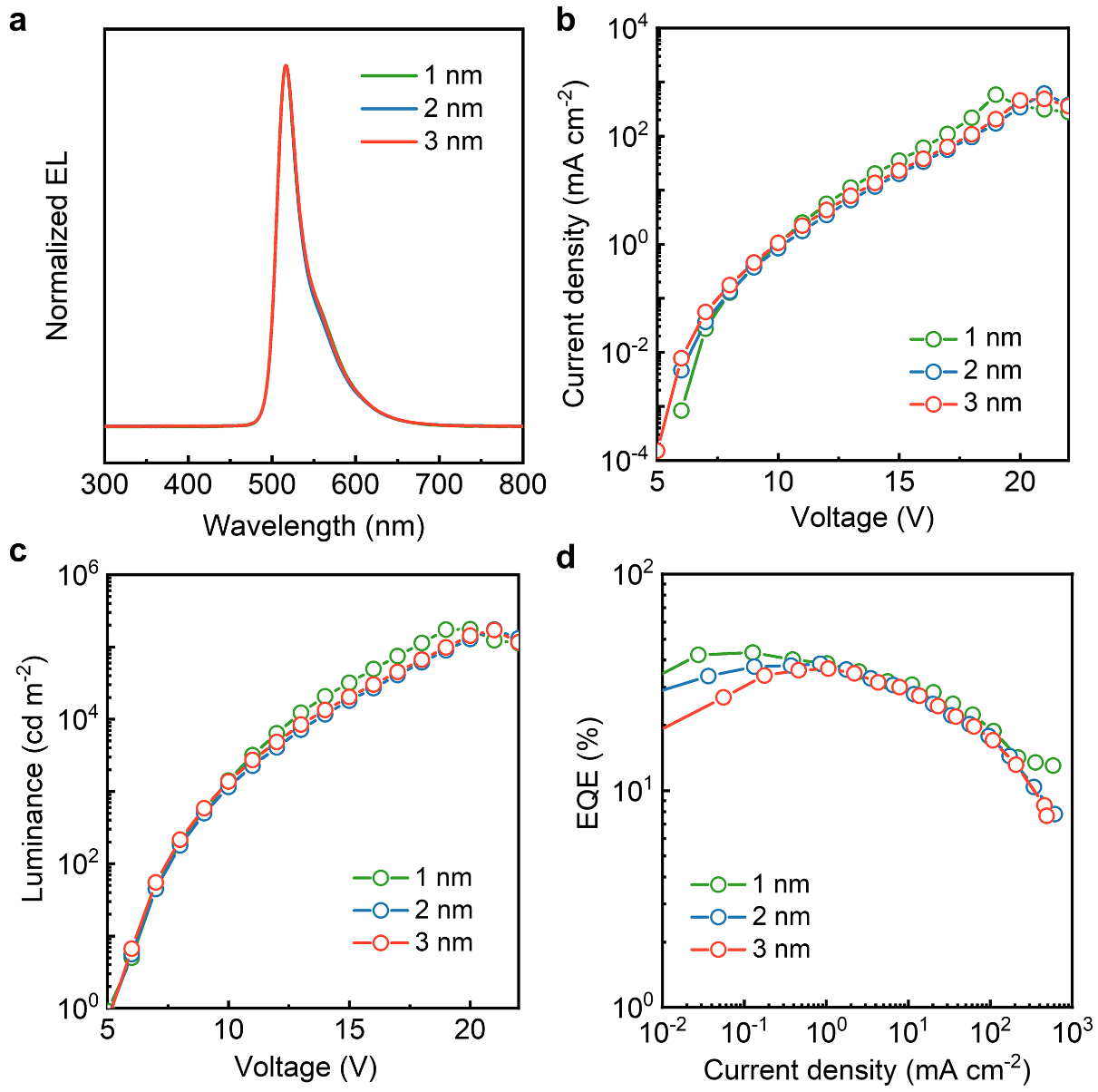
**

**Fig. S2.** **a** EL spectra, **b** *J-V,* **c** *L*-*V*, and **d** EQE*-J* characteristics of the hybrid LEDs with varying MoO_3_ thicknesses.

**
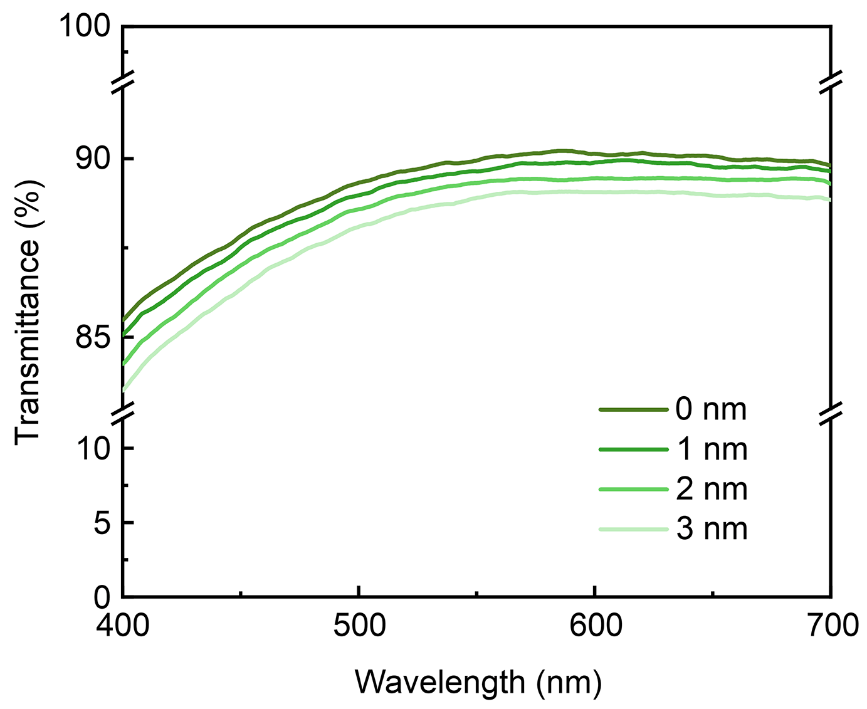
**

**Fig. S3.** Transmittance characteristics of the ICL with varying MoO_3_ thicknesses.


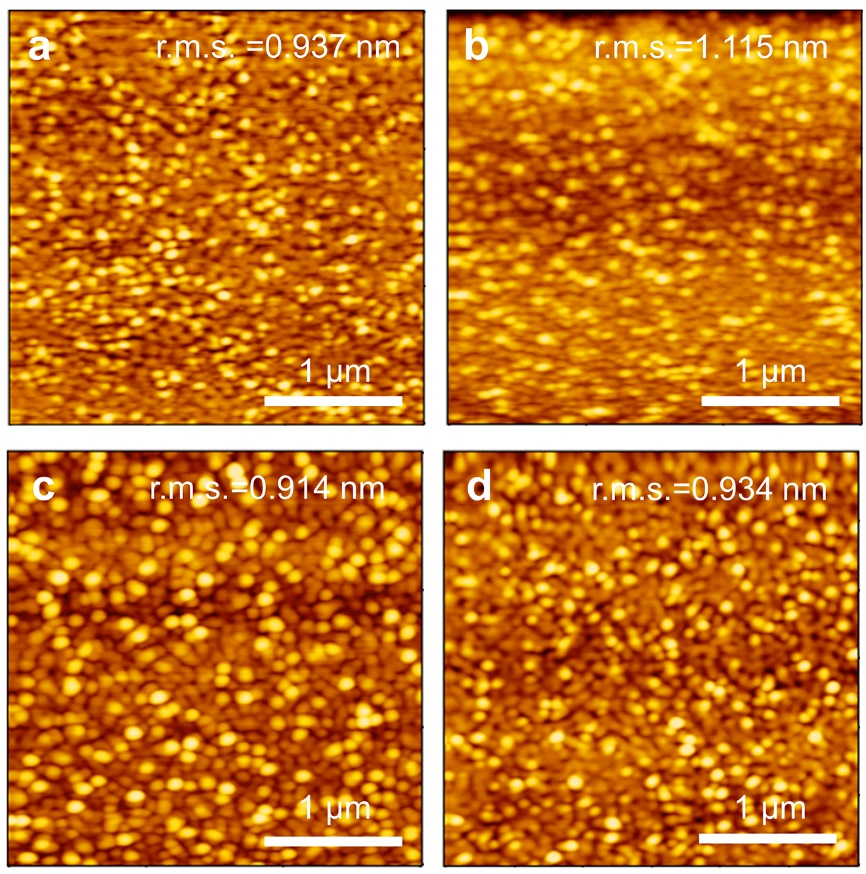


**Fig. S4.** AFM images of HAT-CN films with depositing MoO_3_ for varying thicknesses: **a** 0 nm, **b** 1 nm, **c** 2 nm and **d** 3 nm.


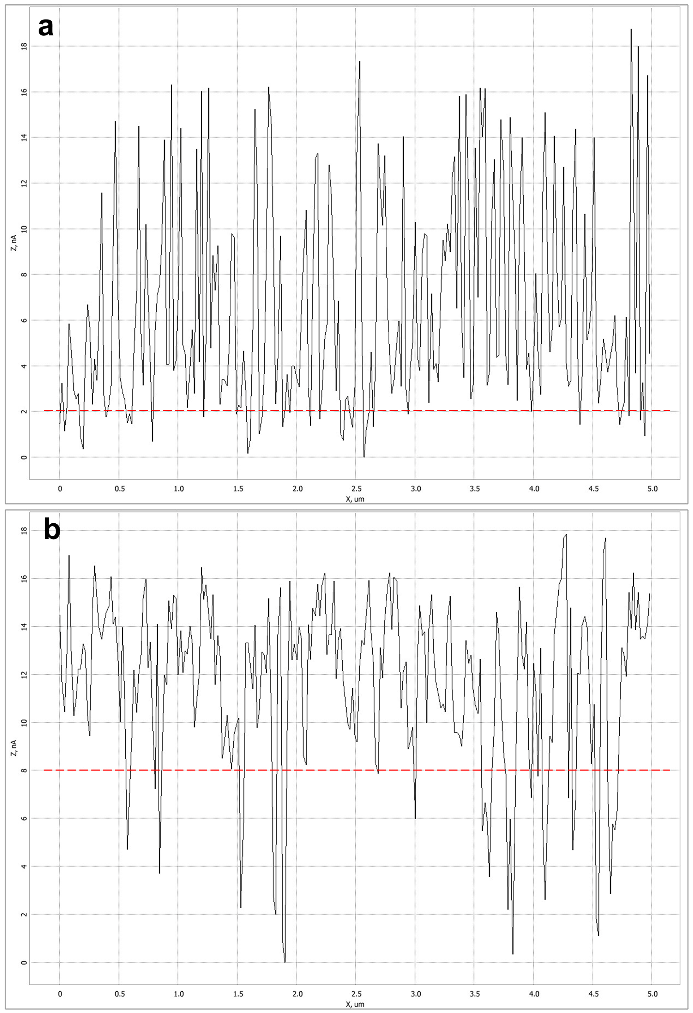


**Fig. S5.** Current profile of **a** HAT-CN and **b** HAT-CN/MoO_3_ films.

**
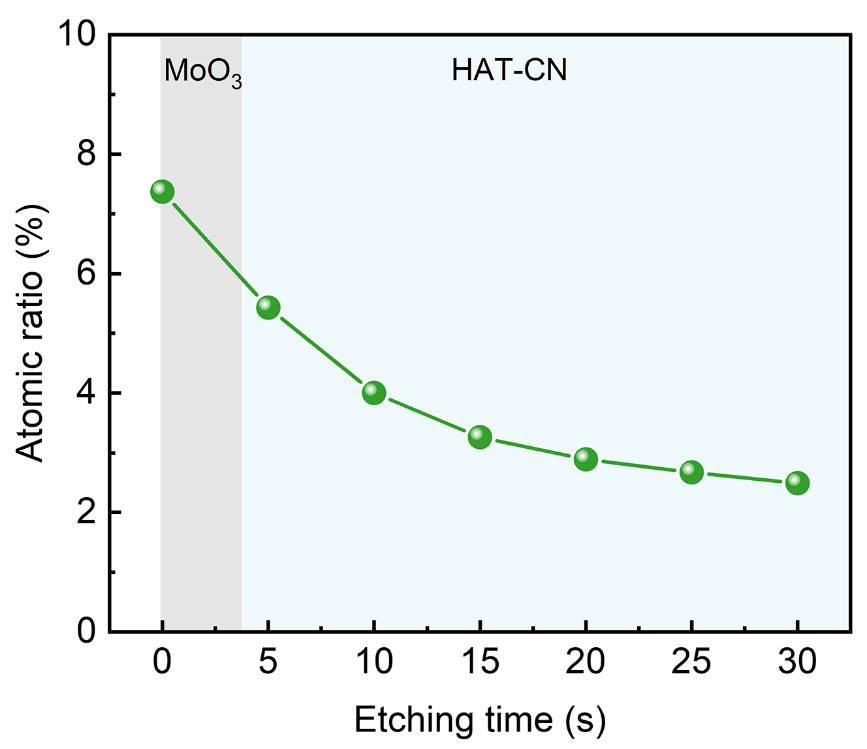
**

**Fig. S6.** Atomic ratios of Mo elements with etching time.

**
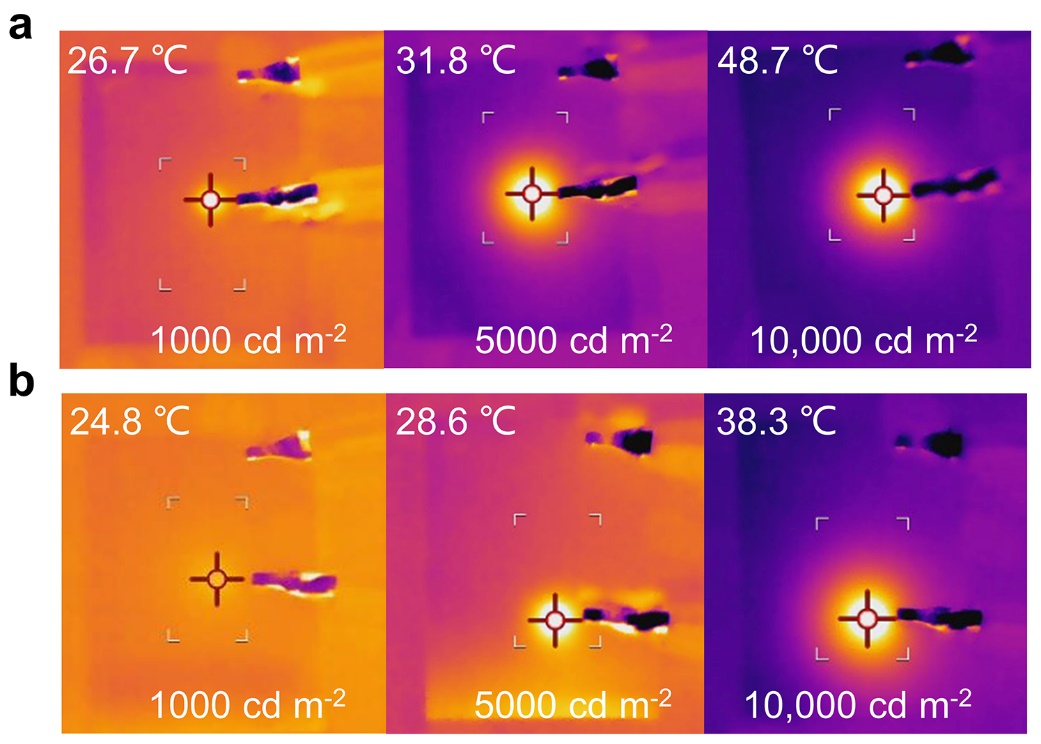
**

**Fig. S7.** Surface temperature of the hybrid LEDs with **a** CGL and **b** m-CGL at different luminance.

**
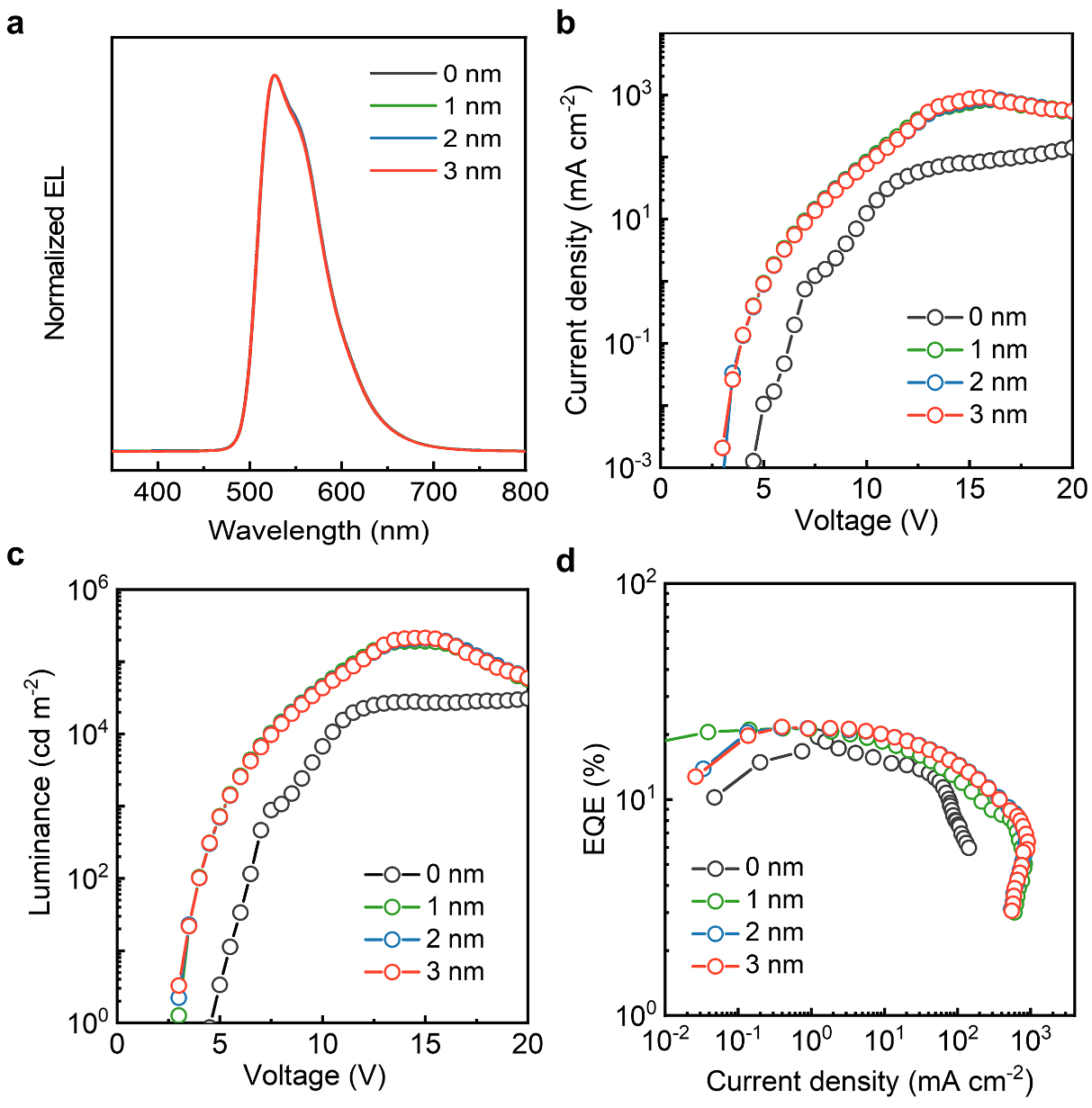
**

**Fig. S8.** **a** EL spectra, **b** *J-V,* **c** *L*-*V*, and **d** EQE*-J* characteristics of the OLEDs with varying MoO_3_ thicknesses.


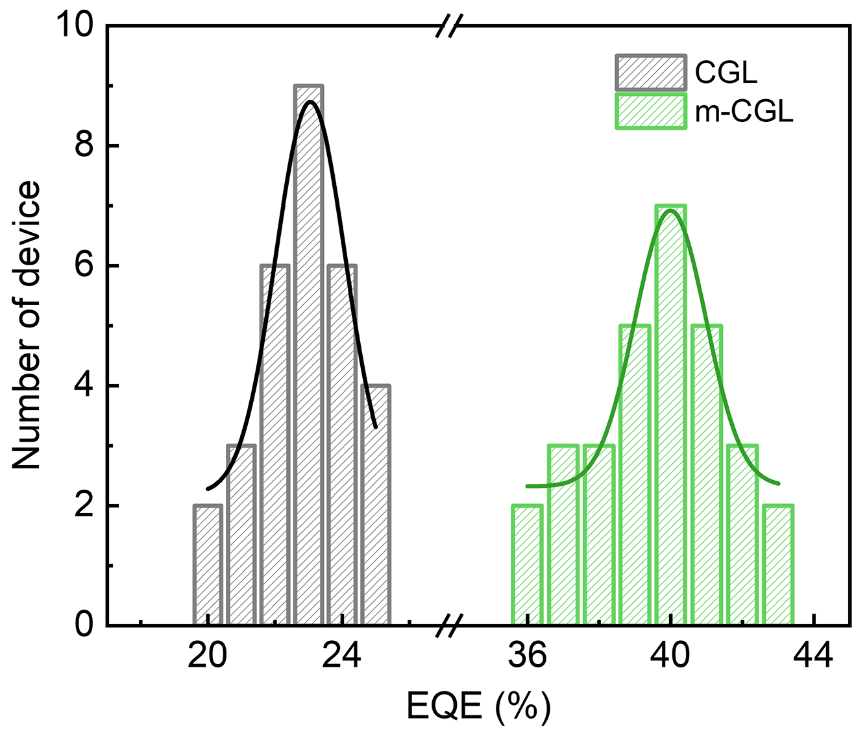


**Fig. S9.** Statistical max. EQE for respective 30 devices with CGL and m-CGL.


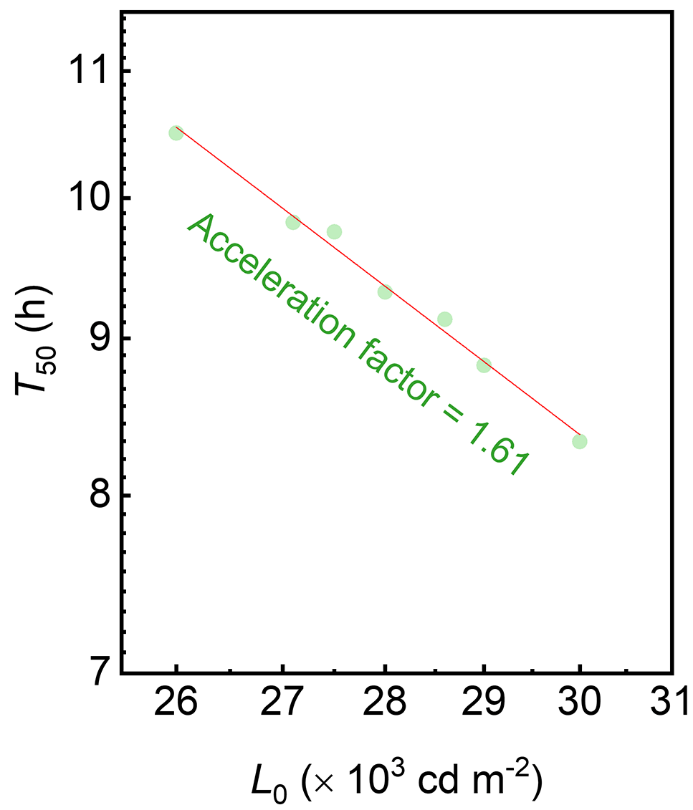


**Fig. S10.** An acceleration lifetime test plot of the hybrid LEDs.

**
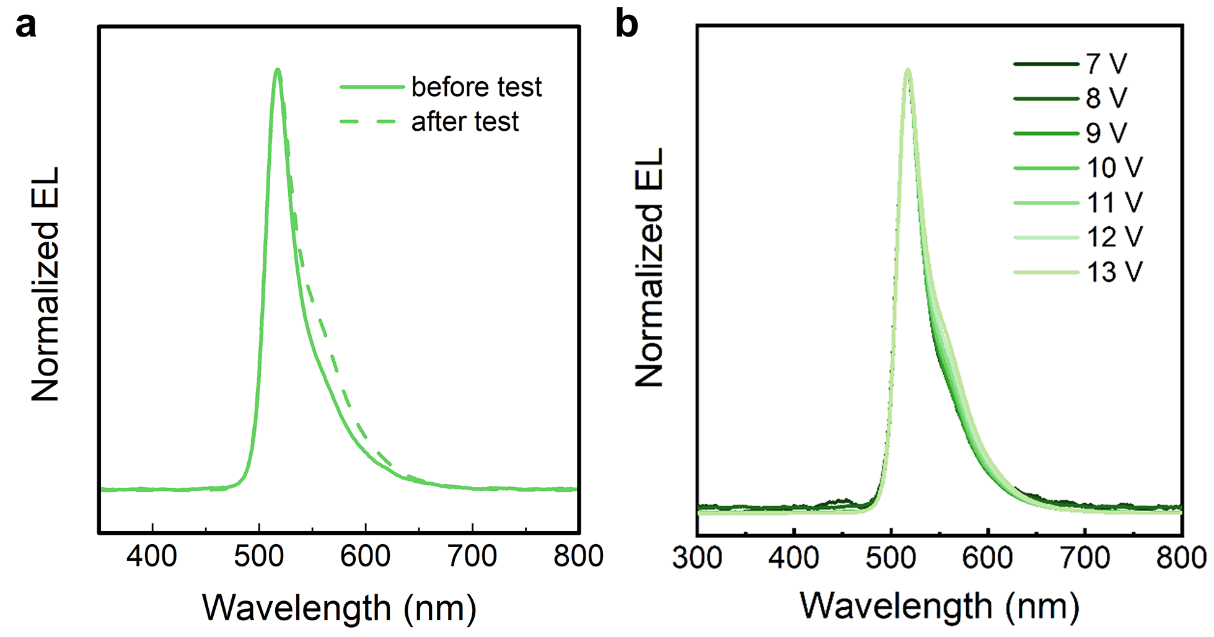
**

**Fig. S11.** Normalized EL spectra of the hybrid devices with m-CGL **a** before and after lifetime test and **b** under continuous voltages.

**
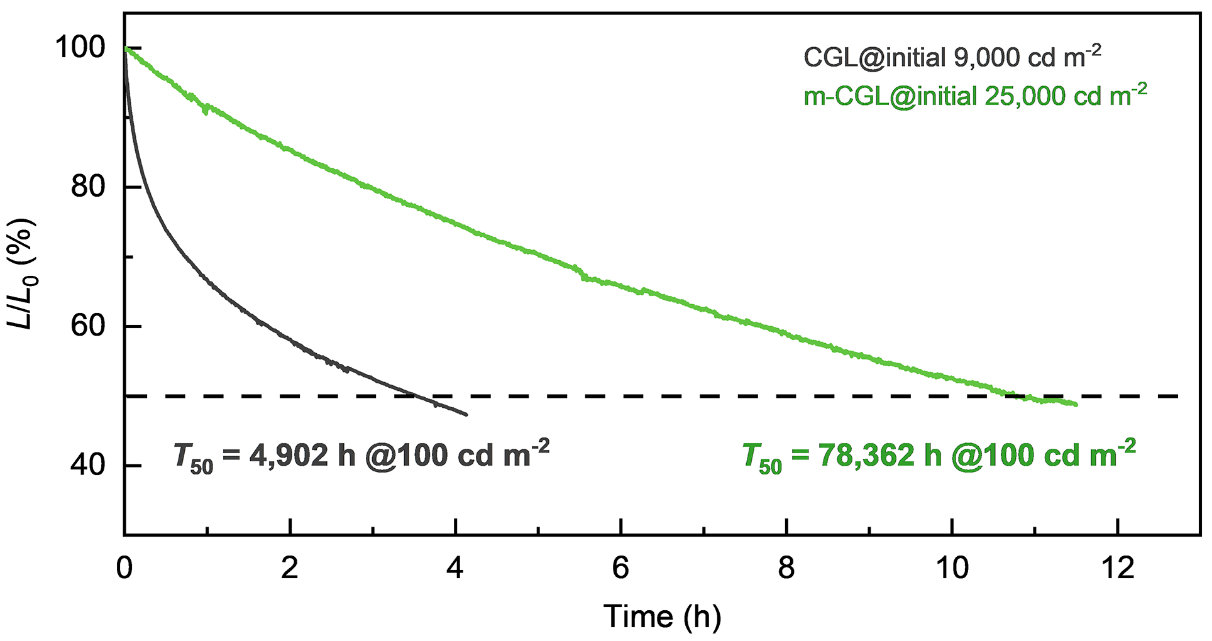
**

**Fig. S12.** Operational stability of the hybrid LEDs based on 3D PeLED and OLED.


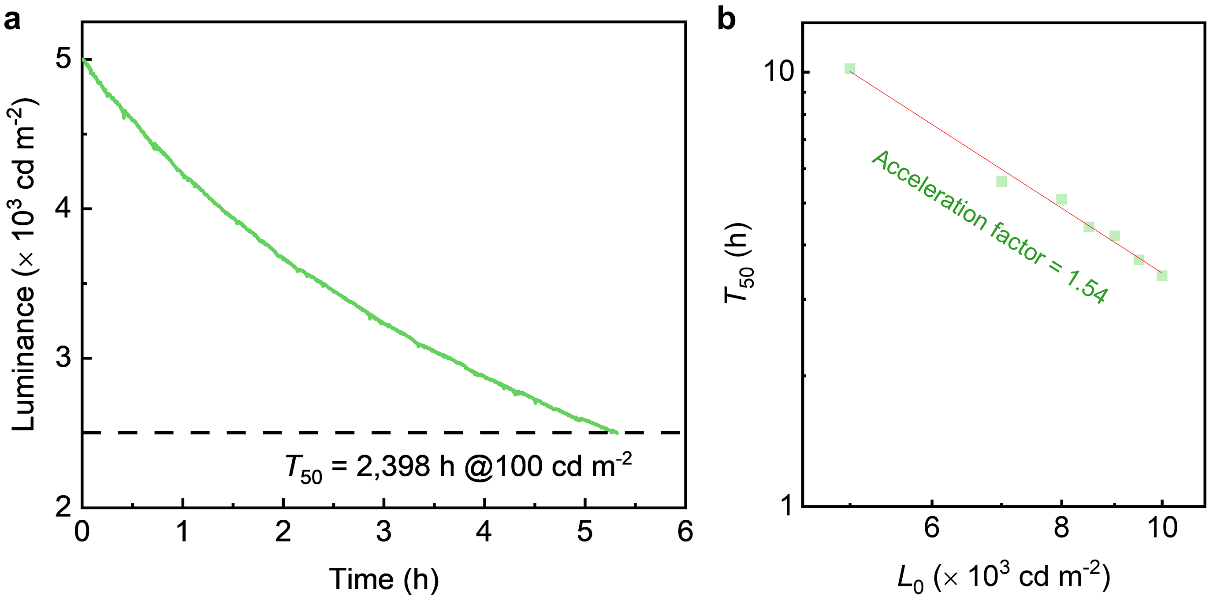


**Fig. S13. a** Operational stability and **b** an acceleration lifetime test plot of the 3D PeLEDs.

**Table S1**. Device performance of the PeLED, OLED and hybrid LED.

| Sample | FWHM  [nm] | EL peak  [nm] | *V*_on_  [V] | Max. Lum.  [cd m^-2^] | Max. CE  [cd A^-1^] | Max. EQE  [%] |
| --- | --- | --- | --- | --- | --- | --- |
| PeLED | ~20 | 514 | ~2.6 | 16,800 | 69.89 | 21.07 |
| OLED | ~67 | 523 | ~3.0 | 191,632 | 77.69 | 21.33 |
| Hybrid LED | ~31 | 516 | ~5.0 | 176,166 | 155.86 | 43.42 |

**Table S2**. Device performance of the hybrid LEDs with varying MoO_3_ thicknesses.

| Sample | FWHM  [nm] | EL peak  [nm] | *V*_on_  [V] | Max. Lum  [cd m^-2^] | Max. CE  [cd A^-1^] | Max. EQE  [%] |
| --- | --- | --- | --- | --- | --- | --- |
| 1 nm | ~31 | 516 | ~5.0 | 176,166 | 155.86 | 43.42 |
| 2 nm | ~31 | 516 | ~5.0 | 174,818 | 136.96 | 38.55 |
| 3 nm | ~31 | 516 | ~5.0 | 171,687 | 130.09 | 36.59 |

**Table S3**. Device performance of the hybrid LEDs with CGL and m-CGL.

| Sample | FWHM  [nm] | EL peak  [nm] | *V*_on_  [V] | Max. Lum  [cd m^-2^] | Max. EQE  [%] |
| --- | --- | --- | --- | --- | --- |
| CGL | ~42 | 517 | ~8.5 | 94,633 | 25.13 |
| m-CGL | ~31 | 516 | ~5.0 | 176,166 | 43.42 |

**Table S4**. Device performance of the OLED with varying MoO_3_ layer thicknesses.

| Sample | FWHM  [nm] | EL peak  [nm] | *V*_on_  [V] | Max. Lum.  [cd m^-2^] | Max. CE  [cd A^-1^] | Max. EQE  [%] |
| --- | --- | --- | --- | --- | --- | --- |
| 0 nm | ~67 | 523 | ~3.0 | 30,664 | 71.77 | 19.51 |
| 1 nm | ~67 | 523 | ~3.0 | 191,632 | 77.69 | 21.33 |
| 2 nm | ~67 | 523 | ~3.0 | 208,691 | 77.90 | 21.50 |
| 3 nm | ~67 | 523 | ~3.0 | 213,945 | 78.24 | 21.67 |
